# Confluent tracheal recurrences of head and neck squamous cell carcinoma

**DOI:** 10.1002/ccr3.2371

**Published:** 2019-08-16

**Authors:** Benjamin Aaron Bleiberg, Muhanned Abu‐Hijleh, William Moore, Saad A. Khan

**Affiliations:** ^1^ Division of Hematology and Oncology, Department of Medicine University of Texas Southwestern Medical Center Dallas Texas; ^2^ Division of Pulmonary and Critical Care Medicine, Department of Internal Medicine University of Texas Southwestern Medical Center Dallas Texas; ^3^ Department of Radiology University of Texas Southwestern Medical Center Dallas Texas

**Keywords:** endobronchial metastases, head and neck cancer, proton therapy, squamous cell cancer

## Abstract

Tracheal head and neck squamous cell cancer recurrence without metastases may be related to physical displacement of cancer cells.

## 1 INTRODUCTION TO CASE REPORTS

We report two patients with unique recurrences of head and neck squamous cell cancer (HNSCC) who were disease‐free at the primary site after surgery and chemoradiation. They then developed recurrences matching the primary cancer along the entire tracheal length but without other distant metastases, suggesting possible novel mechanisms of spread.

Head and neck squamous cell cancer (HNSCC) has a propensity to recur in the neck or locally, and are routinely managed with salvage surgery. If surgery is not possible, systemic therapy approaches including chemotherapy and immune therapy are used but are not curative. Herein, we present the cases of two distinct patients with HNSCC who were treated using standard approaches yet presented with extremely focal recurrences that were confluent across the lumen of the trachea but not at any other point.

After successful surgery and chemoradiation for their primary lesion, both patients developed cancer recurrence along the entire tracheal length without distant metastases. Established protocols for treatment of this type of recurrence may consider this as distant hematogenous metastases; however, novel definitive chemoradiation therapeutic regimens were used with curative results possible. Isolated endotracheal recurrence remains so rare that only five such cases have been reported over the last 2 decades.^1^, ^2^

### 1.1 Case 1

A 56‐year‐old nonsmoking man presented with a left anterior ethmoidal sinus mass. Computed tomography (CT) scan found a T3N1M0 p16‐negative poorly differentiated papillary squamous cell carcinoma. Fludeoxyglucose positron emission tomography (FDG‐PET) scan identified a left nasal cavity mass with osseous destruction and asymmetric left level 2 adenopathy but no disease outside the neck. The tumor was a highly vascular, friable abnormal mass that invaded the lamina papyracea. It extended into the posterior frontal recess and lateral aspect of middle turbinate superiorly and could not be fully resected. After partial endoscopic resection, the patient underwent chemoradiation with 6 weeks cisplatin (40 mg/m^2^) and radiation therapy (72 Gy total dose). Initially, he tolerated it well, but later developed pain and significant weight loss.

**Figure 1:**
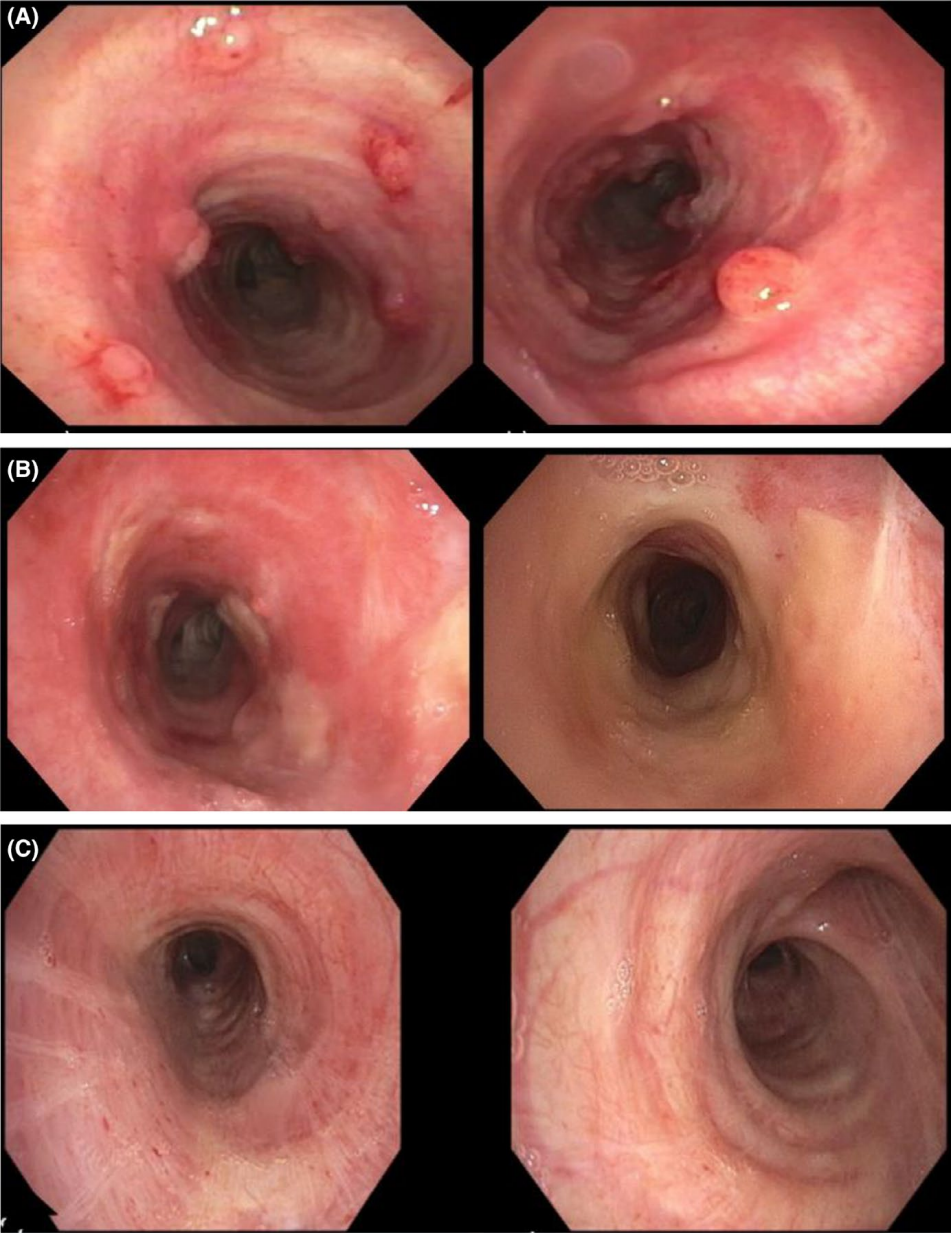
Bronchoscopic appearance of endotracheal metastases that developed after definitive treatment. A, demonstrating evidence of multifocal sessile polypoid lesions in the trachea with variable sizes ranging from 2 mm to 15 mm. B, showing response of lesions after repeated directly ablation cryotherapy and argon plasma coagulation (left); and then shortly after proton therapy with chemotherapy (right). C, shows bronchoscopic no evidence of disease 5 mo after proton therapy

After the completion of his chemoradiation, follow‐up PET imaging demonstrated a significant decrease in the size and prominence of the left anterior ethmoid mass and decreased enhancement in the floor of the left maxillary sinus. One year after chemoradiation, papillary recurrences on the tracheal rings on the mucosal surface were identified on direct evaluation (Figure 1A). The patient underwent three endoscopic cryoablations for multiple squamous cell carcinomas of the tracheal rings, extending throughout the trachea. These tumors shared the cytologic, morphologic, and p16‐negative features of the original tumor. Several weeks to months after each endoscopic cryoablation, recurrent tracheal squamous cell carcinomas were identified, but CT imaging of the neck and chest showed no other area of cancer involvement. These instances of recurrent disease extended on the mucosal surface from the patient's subglottis to the distal trachea, increasing the risk of complications.

As the disease was limited to the trachea without lung parenchymal metastases, the mechanism of metastasis was suspected to be direct mechanical spread (possibly instrumentation or inhalation‐related) during the peri‐operative period following the resection of the patient's primary lesion. Another possible explanation is that this was isolated hematogenous spread sustained over many months. After each tracheal squamous cancer procedure, he was left with no measurable disease and declined systemic chemotherapy. He was offered intensity‐modulated radiation to the trachea which he declined. He did accept curative intent proton therapy (cGyRBE 5400/6400) with concurrent weekly paclitaxel (30 mg/m^2^) for his extensive tracheal recurrences. PET imaging performed 5 months after completion of his last proton therapy showed no lung metastases and no FDG avid areas in the trachea. The patient remains without evidence of disease on bronchoscopic and radiographic evaluation 12 months after completion of all chemo and proton therapy (Figure 1C).

### 1.2 Case 2

A 67‐year‐old nonsmoking man presented with headaches and dizziness with concurrent dysphagia and right otalgia for 1 week. Head and neck CT incidentally identified a hypermetabolic right tonsil mass lesion extending across midline to the left palatine tonsil involving the tongue base and soft palate. PET‐CT identified bilateral FDG avid, multiloculated cystic hyper‐enhancing cervical lymph nodes. He was diagnosed with stage IVa T2N2aM0 p16‐positive squamous cell carcinoma.

**Figure 2:**
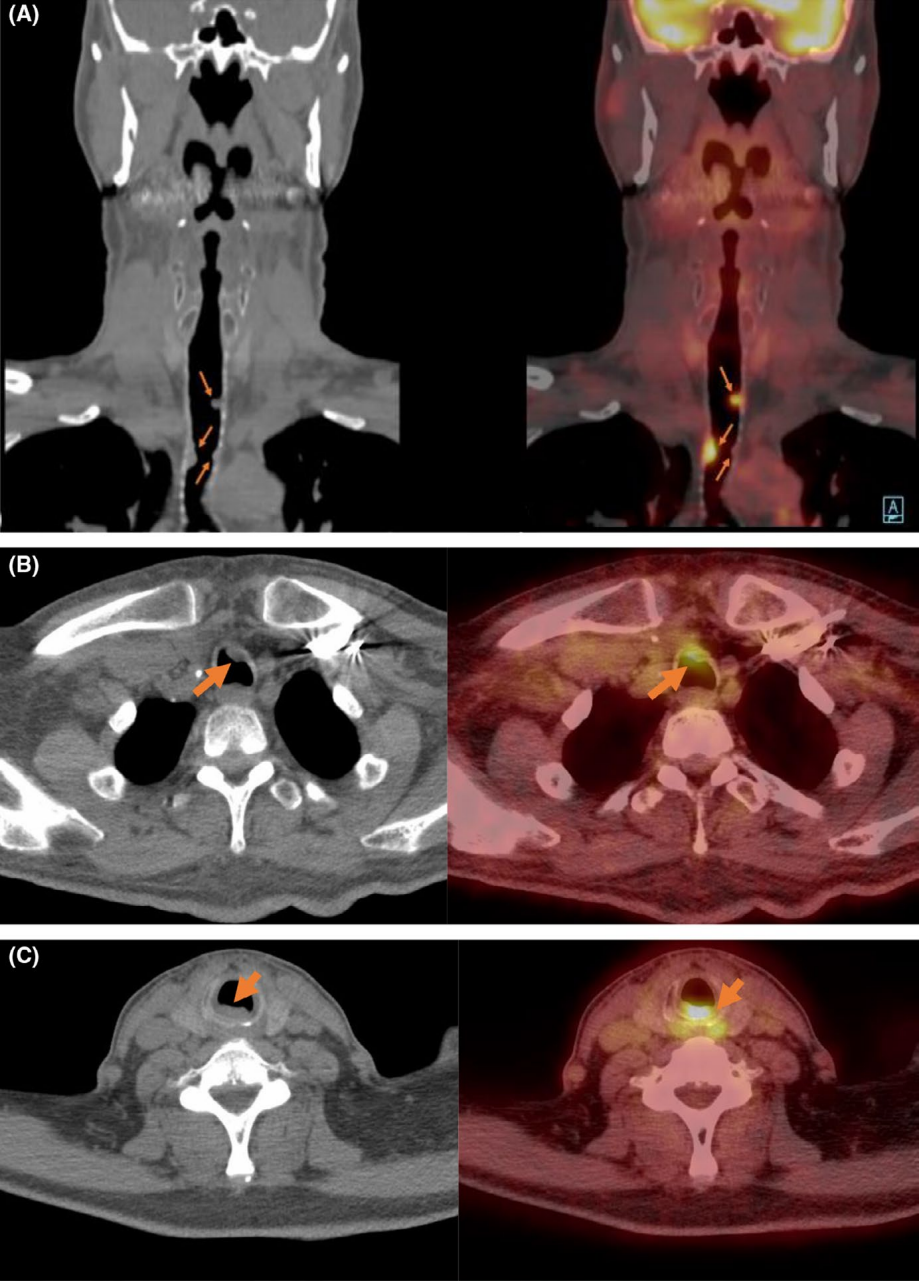
Radiographic development of intra‐luminal endobronchial metastases: A, interval development of multifocal FDG avid (SUV max 9.2) nodular soft masses on CT (left) and FDG‐PET (right) at the tracheal surface of patient 1. B, C, from Patient 2 showing CT and FDG‐PET lesions involving the subglottic larynx and ventral trachea

Following partial resection, he was treated with cetuximab for 6 weeks (loading dose 400 mg/m^2^ then weekly dose of 250 mg/m^2^) and concurrent radiation therapy (total dose of 69.96 Gy), which he completed despite significant nausea and weight loss. Post‐treatment PET scan demonstrated subtotal resolution of the soft tissue in the suprahyoid of the neck and cervical nodes. However, 9 months post‐treatment, a PET imaging showed FDG avid soft tissue thickening in the posterior wall of the subglottic larynx and in a long segment of the anterior tracheal wall extending from the thoracic inlet to the carina (Figure 2B). Repeat chest CT showed sessile and exophytic nodules involving the membranous and cartilaginous portions of the trachea (Figure 2B). Repeat neck CT showed soft tissue thickening of the posterior subglottic tracheal wall and nodular and crescentic foci of thickening in the anterior tracheal wall corresponding to FDG avidity, without signs of recurrence in the region of the right tonsil (Figure 2C).

The patient underwent salvage surgery remove his pedunculated papillomatous laryngeal and tracheal lesions. These masses appeared malignant on histology, but did not demonstrate invasion of the basement membrane, which is typical of this variant. The recurrences in the anterior and posterior trachea were classified on biopsy as diffuse, persistent, unresectable papillary squamous cell carcinomas—which were nearly identical to the primary tonsillar lesion.

Follow‐up imaging demonstrated continuing spread from the confluent crescentic lesions on the anterior wall of the trachea. There were no lung lesions noted or disease outside the trachea. A year after completion of radiation to the oropharynx and involved neck, he was given 69.96 Gy of radiation to the larynx and trachea. He was offered cisplatin concurrently but his performance status declined to the point that he was unable to tolerate more than 2 attenuated doses and completed the remainder of his treatment while hospitalized. Several months after his radiation, he developed pulmonary nodules consistent with pulmonary metastases.

## 2 DISCUSSION

Isolated endobronchial head and neck metastases with no lung parenchymal involvement were originally described as rare, occurring in 1/16 patients with various cancers.^1^, ^2^ A recent report suggested isolated endobronchial recurrences from head and neck squamous cancer in p16 + and p16 negative patients^3^. Here, we reported atypical HNSCC recurrences which were confluent over the entire length of the trachea without any concurrent evidence of distant metastases.

Isolated endobronchial HNSCC metastases are unique because they may represent novel patterns of spread and require creative, curative treatment approaches. Endobronchial metastases from other primary sites such as breast or colon primaries are presumed to spread hematogenously, and systemic therapy is reasonable. HNSCC creeping along the length of the trachea has an ambiguous mechanism of disease spread from the primary site. Possible mechanisms include inhalation or instrumentation during the peri‐operative period resulting in mechanical movement of viable cells into the trachea, where they became embedded in the mucosa and continued to grow. This mechanism of spread is analogous to implantation metastases of the skin from biopsy needles or surgical instruments. “Aerial” metastases to the lung from inhalation of laryngeal papillomas were proposed as early as 1932, which presents a similar mechanism to this presentation, in which the recurrences extend across large fields of the mucosal surface from the thoracic inlet to the carina.^4^

While cryotherapy ablation temporarily managed discrete recurrent lesions in one of the patients described, it was not an adequate curative treatment option. For our patients with tracheal spread of the cancer, chemotherapy or immune therapy would have been offered for both patients as noncurative, alternate standard treatment. For the first patient, proton therapy was delivered with curative intent along the extent of the trachea with concurrent chemotherapy and has resulted in durable disease control without the addition of any subsequent systemic therapy or visible recurrence. Proton therapy was proposed to reduce the toxicity of radiation to adjoining normal tissue,^5^, ^6^ and the patient agreed to undergo such therapy. The outcome for this patient was excellent though he remains in observation with no evidence of disease. For the other patient, a similar approach succeeded in disease control in the trachea, but was followed by subsequent development of metastatic disease and poor response to systemic therapy. A significant finding that in the absence of systemic therapy, no distant metastases outside the trachea have developed in our first patient who completed optimal chemoradiation to the tracheal recurrence, while the distant metastases did develop in the second patient who was unable to complete his tracheal chemoradiation.

These two cases highlight a rare type of atypical HNSCC regional recurrence but defy easy explanation or categorization. Cancer primary site, p16 status, initial treatments offered as well as subsequent outcome all differed significantly, with no obvious common connection. Alternate approaches to directly ablative or radiation‐based approaches include an initial trial of systemic therapy, though currently poor response rates and the noncurative nature of these treatments make them less attractive to patients.

## 3 CONCLUSIONS

Isolated endotracheal recurrences of HNSCC can occur in both p16 positive and p16 negative cancers arising from various primary sites and should be managed by a multidisciplinary team taking into account the potential for cure with directly ablative and tracheal radiation approaches. Mechanical manipulation of tumors may have contributed to the subsequent development of endotracheal metastases, though unique cancer biology may have contributed. We recommend extra care to limit mechanical manipulation of tumors during office procedures or in the operating room. Patients with isolated endotracheal recurrences can be cured with radiation therapy, including proton therapy approaches.

## CONFLICTS OF INTEREST

None.

## AUTHOR CONTRIBUTIONS

Mr Benjamin Aaron Bleiberg: contributed to concept for manuscript, majority of writing, and critical review of manuscript. Dr Muhanned Abu‐Hijleh: involved in critical review and editing of manuscript, generation of clinical findings in patient via direct care, and final review of manuscript. Dr William Moore: involved in critical review and editing of manuscript, generation of clinical findings in patient via direct care, and final review of manuscript. Dr Saad A. Khan: contributed to concept for manuscript, critical review and editing of manuscript, generation of clinical findings in patient via direct care, and approval of final draft of manuscript.
